# Using fMRI Brain Activation to Identify Cognitive States Associated with Perception of Tools and Dwellings

**DOI:** 10.1371/journal.pone.0001394

**Published:** 2008-01-02

**Authors:** Svetlana V. Shinkareva, Robert A. Mason, Vicente L. Malave, Wei Wang, Tom M. Mitchell, Marcel Adam Just

**Affiliations:** 1 Department of Psychology, Carnegie Mellon University, Pittsburgh, Pennsylvania, United States of America; 2 Machine Learning Department, School of Computer Science, Carnegie Mellon University, Pittsburgh, Pennsylvania, United States of America; Indiana University, United States of America

## Abstract

Previous studies have succeeded in identifying the cognitive state corresponding to the perception of a set of depicted categories, such as *tools*, by analyzing the accompanying pattern of brain activity, measured with fMRI. The current research focused on identifying the cognitive state associated with a 4s viewing of an individual line drawing (1 of 10 familiar objects, 5 *tools* and 5 *dwellings*, such as a *hammer* or a *castle*). Here we demonstrate the ability to reliably (1) identify which of the 10 drawings a participant was viewing, based on that participant's characteristic whole-brain neural activation patterns, excluding visual areas; (2) identify the category of the object with even higher accuracy, based on that participant's activation; and (3) identify, for the first time, both individual objects and the category of the object the participant was viewing, based only on other participants' activation patterns. The voxels important for category identification were located similarly across participants, and distributed throughout the cortex, focused in ventral temporal perceptual areas but also including more frontal association areas (and somewhat left-lateralized). These findings indicate the presence of stable, distributed, communal, and identifiable neural states corresponding to object concepts.

## Introduction

It has been a lasting challenge to establish the correspondence between a simple cognitive state (such as the thought of a *hammer*) and the underlying brain activity. Moreover, it is unknown whether the correspondence is the same across individuals. A recent approach to studying brain function uses machine learning techniques to identify the neural pattern of brain activity underlying various thought processes. Previous studies using a machine learning approach have been able to identify the cognitive states associated with viewing an object category, such as houses [1], [2], [3], [4], [5], [6], [7], [8]. The central characteristic of this approach (compared to a conventional statistical parametric mapping-like approach) is its identification of a multivariate pattern of voxels and their characteristic activation levels that collectively identify the neural response to a stimulus. These machine learning methods have the potential to be particularly useful in uncovering how semantic information about objects is represented in the cerebral cortex because they can determine the topographic distribution of the activation and distinguish the content of the information in various parts of the cortex. In the study reported below, the neural patterns associated with individual objects as well as with object categories [9] were identified using a machine learning algorithm applied to activation distributed throughout the cortex. This study also investigated the degree to which objects and categories are similarly represented neurally across different people.

**Figure 1:**
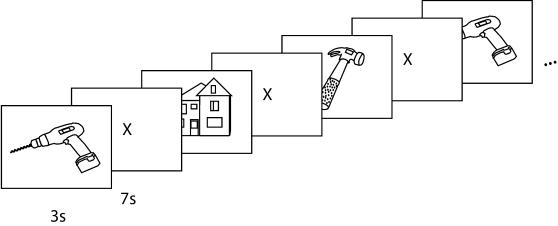
Schematic depiction of presentation timing.

We analyzed the brain activity of participants who were viewing a line drawing of an object from the categories of *tools* or *dwellings*, of the type shown in Figure 1. We were able to train classifiers to identify which of ten object exemplars and two object categories a participant was viewing. We discovered a common neural pattern across participants, and used this to train a classifier to identify the correct object category and object exemplar from the fMRI data of new participants who were not involved in training the classifier.

## Materials and Methods

### Participants

Twelve right-handed adults (8 female) from the Carnegie Mellon community participated and gave written informed consent approved by the University of Pittsburgh and Carnegie Mellon Institutional Review Boards. Six additional participants were excluded from the analysis due to head motion greater than 2.5 mm.

### Experimental paradigm

The stimuli depicted concrete objects from two semantic categories (*tools* and *dwellings*), and took the form of white line drawings on a black background. There were five exemplars per category; the objects were *drill*, *hammer*, *screwdriver*, *pliers*, *saw*, *apartment*, *castle*, *house*, *hut*, and *igloo*. The drawings of the ten objects were presented six times (in six random permutation orders) to each participant. Participants were asked to think of the same object properties each time they saw a given object, to encourage activation of multiple attributes of the depicted object, in addition to those used for visual recognition. The intention was to foster the retrieval and assessment of the most salient properties of an object. To ensure that each participant had a consistent set of properties to think about, he or she was asked to generate a set of properties for each exemplar prior to the scanning session (such as *cold*, *knights*, and *stone* for *castle*). However, nothing was done to elicit consistency across participants.

Each stimulus was presented for 3s, followed by a 7s rest period, during which the participants were instructed to fixate on an X displayed in the center of the screen. There were six additional presentations of a fixation X, 21s each, distributed across the session to provide a baseline measure of activation. A schematic representation of the presentation timing is shown in Figure 1.

### fMRI procedure

Functional images were acquired on a Siemens Allegra 3.0T scanner (Siemens, Erlangen, Germany) at the Brain Imaging Research Center of Carnegie Mellon University and the University of Pittsburgh using a gradient echo EPI pulse sequence with TR = 1000 ms, TE = 30 ms, and a 60° flip angle. Seventeen 5-mm thick oblique-axial slices were imaged with a gap of 1 mm between slices. The acquisition matrix was 64×64 with 3.125×3.125×5 mm^3^ voxels.

### fMRI data processing and analysis

Data processing and statistical analysis were performed with Statistical Parametric Mapping software (SPM99, Wellcome Department of Imaging Neuroscience, London, UK). The data were corrected for slice timing, motion, linear trend, and were temporally smoothed with a high-pass filter using a 190 s cutoff. The data were normalized to the Montreal Neurological Institute (MNI) template brain image using a 12-parameter affine transformation. Group contrast maps were constructed using a height threshold of p<0.001 (uncorrected) and an extent threshold of 160 voxels, resulting in the cluster-level threshold of p<0.05, corrected for multiple comparisons.

Analyses of a single brain region at a time used region definitions derived from the Anatomical Automatic Labeling (AAL) system [10]. In addition to existing AAL regions, left and right intraparietal sulcus (IPS) regions were defined, and superior, middle, and inferior temporal gyrus regions were separated into anterior, middle, and posterior sections based on planes F and D from the Rademacher scheme [11], for a total of 71 regions.

The data were prepared for machine learning methods by spatially normalizing the images into MNI space and resampling to 3×3×6 mm^3^ voxels. Voxels outside the brain or absent from at least one participant were excluded from further analysis. The percent signal change (PSC) relative to the fixation condition was computed at each voxel for each object presentation. The mean PSC of the four images acquired within a 4s window, offset 4s from the stimulus onset (to account for the delay in hemodynamic response) provided the main input measure for the machine learning classifiers. The PSC data for each object-presentation were further normalized to have mean zero and variance one to equalize the between-participants variation in exemplars.

### Machine learning methods

Classifiers were trained to identify cognitive states associated with viewing drawings, using the evoked pattern of functional activity (mean PSC). Classifiers were functions *f* of the form: *f: mean_PSC→Y_j_*, *j* = 1, …, *m*, where *Y_j_* were either categories (*tools*, *dwellings*) or ten exemplars (*hammer*, *pliers*, …, *house*), where *m* was either 2 or 10, accordingly, and where *mean_PSC* was a vector of mean PSC voxel activations. To evaluate classification performance, trials were divided into disjoint training and test sets. Prior to classification, relevant features (voxels) were extracted (as described below) to reduce the dimensionality of the data, using only the training set for this selection. A classifier was built from the training set, using these selected features. Classification performance was then evaluated on only the left-out test set, to ensure unbiased estimation of the classification error. Our previous exploration indicated that several feature selection methods and classifiers produce comparable results. Here we report results from one feature selection method and one classifier, chosen for simplicity.

### Feature selection

Feature selection first identified the voxels whose responses were the most stable over six presentations of objects within a participant, and then selected from among the stable voxels those that best discriminated among objects within the training set, using only the data in the training set. The 400 most stable voxels were selected, where voxel stability was computed as the average pairwise correlation between 10-object vectors across six presentations. In the second step, all of the stable voxels were assessed for how discriminating they were, by training a logistic regression classifier to discriminate among object exemplars or categories on various subsets of only the training set. Finally, from among the 400 voxels selected for stability, discriminating subsets of sizes 10, 25, 50, 75, 100, 200, and 400 voxels were selected based on having the highest (absolute valued) regression weights in the logistic regression. Locations of these selected voxels (henceforth, diagnostic voxels) were visualized on a standard brain template using MRIcro [12].

### Classification

The Gaussian Naïve Bayes (GNB) pooled variance classifier was used [13]. It is a generative classifier that models the joint distribution of a class *Y* and attributes *X*, and assumes the attributes *X_1_*, …, *X_n_* are conditionally independent given *Y*. The classification rule is:
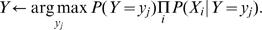
In this experiment classes were equally frequent. Classification results were evaluated using *k*-fold cross-validation, where one example per class was left out for each fold. For each participant, a classifier was trained to identify either which of 10 object exemplars or which of two object categories that participant was viewing, based on only 4 s of fMRI data per object presentation. In all analyses, the accuracy of identification was based only on test data that was completely disjoint from the training data. With a two-class classification problem, the chance level is 0.5. With the ten-class classification problem, *rank accuracy* was used [13]. The list of potential classes was rank-ordered from most to least likely, and the normalized rank of a correct class in a sorted list was computed. Rank accuracy ranges from 0 to 1, and the chance level is 0.5.

Peak classification accuracy over the previously defined subsets having different numbers of voxels, e.g., 10, 25, …, 400, was reported. To evaluate the statistical significance of this observed classification accuracy, the result was compared to a permutation distribution. For each of the 1,000 non-informative permutations of labels in the training set, permutation classification accuracies for every set of features were computed, and the best permutation accuracy over the subsets with different numbers of voxels was recorded. The observed accuracy was then compared to the distribution of recorded permutation classification accuracies; if the observed accuracy had a p-value of at most 0.001, then the result was considered statistically significant.

### Analyses of a single brain region at a time

Single anatomical brain regions that consistently identified object exemplars or categories across participants were selected using cross-validation, and the significance of those identifications was tested across participants. Within each participant, a cross-validated accuracy for each region was computed by a logistic regression classifier using all the voxels from that anatomical region. The mean classification accuracy was computed for each anatomical region across participants, and compared to a binomial distribution. The obtained p-values (computed using a normal approximation) were compared to the level of significance α = 0.001, using the Bonferroni correction to account for the multiple comparisons.

### Analysis of the confusion patterns

Single brain regions were compared in terms of their confusion patterns using a generalization of the principal components analysis method [14], [15]. Within each participant, for each of the selected regions, a confusion matrix was constructed based on the most likely prediction of the classifier. Next, a regions-by-regions dissimilarity matrix was constructed for each participant, where the dissimilarity between any two anatomical regions was measured as one minus the correlation coefficient of the off-diagonal elements of the corresponding confusion matrices. Each dissimilarity matrix was transformed to a cross-product matrix and normalized by the first eigenvalue.

A compromise matrix, representing the agreement across participants, was constructed as a weighted average of all the participants' regions-by-regions cross-product matrices. Participants' weights were computed from the first principal component of the participants-by-participants similarity matrix (the first principal component is proportional to the mean of the participant matrices). Each entry in the participants-by-participants similarity matrix was computed by the RV-coefficient [16], which is a multivariate extension of the Pearson correlation coefficient, and indicates the overall similarity of the two matrices:
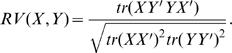
The RV-coefficient has been previously used in the fMRI literature [17], [18]. The compromise matrix was further analyzed by principal components analysis.

### Multiple participant analysis

Data from all but one participant were used to train a classifier to identify the data from the left-out participant. This process was repeated so that it reiteratively left out each of the participants. Feature selection was done by pooling the data of all participants but the one left out. Discriminating voxel subsets of sizes 10, 25, 50, 75, 100, 200, 400, 1000, and 2000 were selected on the basis of logistic regression weights.

## Results

### Identifying object exemplars: whole brain

**Figure 2:**
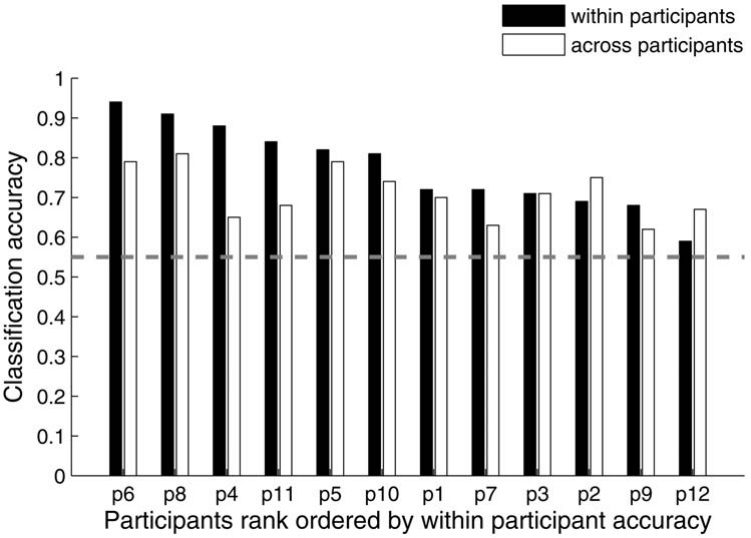
High classification rank accuracies for object exemplars. Reliable (p<0.001) accuracies for the classification of object exemplars within participants (filled bars) were reached for eleven out of twelve participants, and reliable (p<0.001) accuracies for the classification of object exemplars when training on the union of data from eleven participants (unfilled bars) were reached for eight out of twelve participants. The dashed line indicates the highest mean of the permutation distribution across participants under the null hypothesis of no difference, i.e., chance level, among object exemplars for cross-participants object exemplar identification.

The highest rank accuracy achieved for any participant while identifying individual object exemplars was 0.94. (The identification process obtained this rank accuracy by correctly identifying the object on its first-ranked guess in 40 out of 60 presentations, on its second-ranked guess in 10 presentations, and on its third- and fourth-ranked guesses in 10 other presentations.) Reliable (p<0.001) classification accuracy for individual object exemplars was reached for eleven out of twelve participants (as shown by the filled bars in Figure 2). The mean classification rank accuracy over all 12 participants was 0.78 (SD = 0.11).

**Figure 3:**
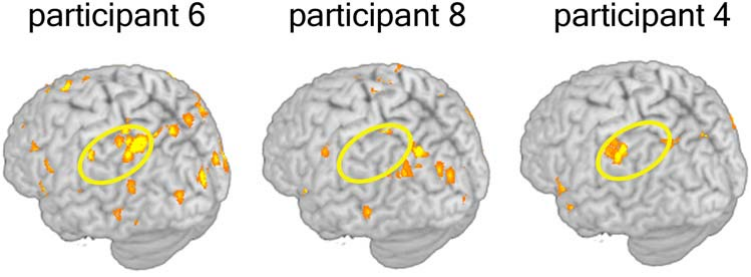
Locations of the diagnostic voxels in object exemplar classification for the three participants having the highest accuracies are shown on the three-dimensional rendering of the T1 MNI single-subject brain. Yellow ellipses indicate the commonality of the voxel locations for object identification in LIPL across participants.

The locations of voxels that underpinned this accurate object exemplar identification (i.e., the diagnostic voxels), were similar (at a gyral level) across participants, and were distributed across the cortex (as shown in Figure 3). They were located in the left inferior frontal gyrus (LIFG), left inferior parietal lobule (LIPL), and bilateral medial frontal gyrus, precentral gyrus, posterior cingulate, parahippocampal gyrus, cuneus, lingual gyrus, fusiform gyrus, superior parietal lobule (SPL), superior temporal gyrus, and middle temporal gyrus. The number of voxels (each 3.125×3.125×6 mm^3^ or 59 mm^3^ in volume) for which object exemplar identification accuracy was greatest (as plotted in Figure 2) ranged from 25 to 400 voxels, depending on the participant (Table S1). (Although the results are reported here for voxel set sizes that have been tuned for individual participants, the results are not substantially different when a fixed set size of voxels is used for item and category classification, within and between participants. For example, for the within-participant identification of individual items, the mean accuracy (over participants) decreases by 2.7% (from 0.78) when a fixed size of 120 voxels is used for all participants. Thus, the optimization of voxel set size is not critical to our main arguments, and a modal fixed value of 120 voxels can provide similar outcomes.)

### Identifying object exemplars: single brain regions

Previous studies have focused on one particular region, the ventral temporal cortex, in an attempt to relate cognitive states to activation patterns in a particular region (e.g., [7]; Sayres, Ress, and Grill-Spector, 2005, in Proceedings of Neural Information Processing Systems). To determine whether it was possible to identify cognitive states on the basis of the activation in only a single brain region, classifiers were trained using voxels from only one anatomical region (such as LIFG) at a time. The accuracies obtained in this ancillary analysis were surprisingly high. For example, for one participant whose object exemplar identification accuracy based on the whole cortex was 0.94, the single-region accuracy was 0.77 for left superior extrastriate (SES), 0.77 for LIPL, and 0.82 for left inferior extrastriate cortex (IES). The regions that generated reliable accuracies across participants in this single-region identification were bilateral SES, IES, calcarine sulcus, fusiform gyrus, IPS, left IPL, posterior superior, middle and inferior temporal gyri, postcentral gyrus, and hippocampus. Thus, many brain regions contain information about the object exemplars.

These analyses provide two important clues about object representations in the cortex. First, they indicate that the discrimination of objects was not just mediated by basic retinotopic representations in the visual cortex, or by eye movements. Other brain areas also carry reliable information about individual *tools* and *dwellings*, demonstrating that the exemplar identification can be based on the neural representations of higher-level facets of the object properties. Second, they indicate that the activation of many regions individually can discriminate among exemplars, thus providing an important clue concerning the neural representations in different regions, which we explore below.

### Identifying object categories: whole brain

**Figure 4:**
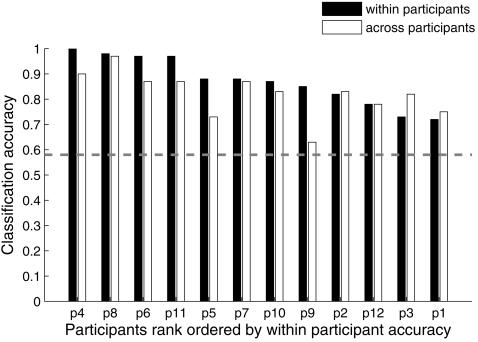
High classification accuracies for object categories. Reliable (p<0.001) accuracies for classification of objects by category (filled bars) were reached for all participants and reliable (p<0.001) accuracies for classification of objects by category when training on the union of data from eleven participants (unfilled bars) were reached for ten out of twelve participants. The dashed line indicates the highest mean of the permutation distribution across participants under the null hypothesis of no difference among the categories (i.e., chance level) for cross-participants category identification.

A classifier was trained to decode which category that the object a participant was viewing belonged to, i.e., whether it was a *tool* or a *dwelling*. Accuracies of at least 0.97 (correct category identification in at least 58 out of 60 object presentations) were attained for four of the participants, including perfect accuracy for one of the participants (correct category identification in 60 out of 60 object presentations) (filled bars in Figure 4). Reliable (p<0.001) classification accuracies were reached for all participants. The mean classification accuracy for category identification across twelve participants was 0.87 (SD = 0.10).

**Figure 5:**
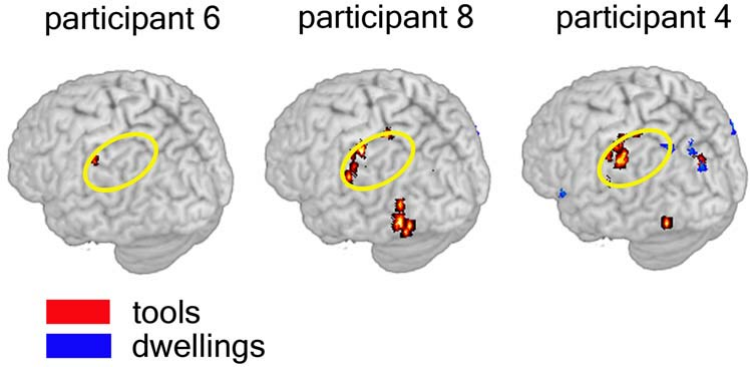
Commonality in voxel locations across the three participants having the highest category classification accuracies. Voxel locations for the *tools* category are shown in red, and voxel locations for the *dwellings* category are shown in blue on the three-dimensional rendering of the T1 MNI single-subject brain. Yellow ellipses indicate commonality in voxel locations for the *tools* category in LIPL across participants.

**Figure 6:**
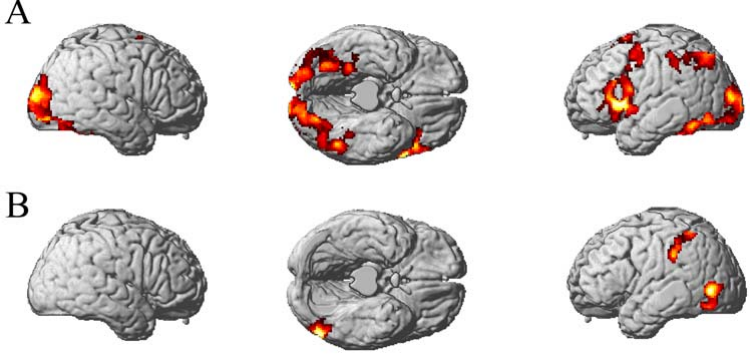
Brain activation showing areas of greater activity for (A) objects compared to fixation, and (B) *tools* compared to *dwellings.* Activation is projected onto a surface rendering.

The locations of the diagnostic voxels were distributed across the cortex. Similarity across participants in the locations of these diagnostic voxels is illustrated in Figure 5. The cortical locations of these voxels provide some face validity for the approach, because they are in areas previously associated with mental functions that bear a good correspondence to the stimuli used here. For example, voxels contributing to the identification of *tools* were mostly in the left hemisphere, and the largest subsets were located in the ventral premotor cortex and posterior parietal cortex. These areas were previously implicated in motor representation associated with tool usage [19], [20], [21]. Some of the voxels contributing to the identification of *dwellings* were located in the right parahippocampal gyrus and were within 9 mm of the previously reported parahippocampal place area (PPA) [22]. The number of voxels for which the object category identification accuracy was greatest ranged from 10 to 100 voxels, depending on the participant (Table S2). For comparison, SPM contrast maps showing areas of greater activity for the objects compared to fixation, and for *tools* compared to *dwellings*, are shown in Figure 6. Similar to the locations of the diagnostic voxels, the activation for *tools* relative to *dwellings* was left-lateralized, and included posterior parietal cortex. In the machine learning analysis, the spatial distribution of the diagnostic voxels was more fine-grained, with some spatial interspersing of voxels between categories, compared to the SPM contrasts.

### Identifying object categories: single brain regions

As was the case for exemplar identification, the accuracies of the category identification using voxels from only a single anatomical region were high; in some cases, these approached the accuracy obtained when the whole cortex was used (0.93 for left IES cortex, 0.83 for left SES cortex, and 0.82 for LIPL, vs. 0.98 for the whole cortex, for one of the participants). The regions that generated reliable accuracies across participants in this single-region identification analysis were bilateral SES, calcarine, IES, SPL, IPL, IPS, fusiform, posterior superior and middle temporal, posterior inferior temporal gyri, cerebellum, and left precentral, superior frontal, inferior frontal triangularis, insula, and postcentral gyri (Table 1). Although the semantic category of the objects can be accurately identified on the basis of a single region, it is even more accurately identified when the whole cortex is taken into account. Similarly to the case of identifying individual object exemplars, reliable information about *tools* and *dwellings* categories resides not only in low-level visual brain areas but also in brain areas that are typically associated with higher-level properties.

**Table 1:** Anatomical regions (out of 71) that singly produced reliable average classification accuracies across the twelve participants for category identification.

Label	Region
LPRECENT	
	L Precentral gyrus
LSUPFRONT	
	L Superior frontal gyrus
LTRIA	
	L Inferior frontal gyrus, triangular part
LINSULA	
	L Insula, rolandic operculum
LCALC, RCALC	
	L/R Calcarine fissure
LSES, RSES	L/R Cuneus, superior occipital, middle occipital gyri
LIES, RIES	
	L/R Inferior occipital, lingual gyri
LFUSIFORM, RFUSIFORM	
	L/R Fusiform gyrus
LPOSTCENT	
	L Postcentral gyrus
LSPL, RSPL	L/R Superior parietal gyrus, precuneus, paracentral lobule
LIPL, RIPL	L/R Inferior parietal, supramarginal, angular gyri
LIPS, RIPS	
	L/R Intraparietal sulcus
LSTPOS, RSTPOS	L/R Posterior superior temporal, posterior middle temporal gyri
LITPOS, RITPOS	
	L/R Posterior inferior temporal gyrus
LCBEL, RCBEL	L/R Cerebellum

L indicates left, and R indicates right hemisphere.

The results above, along with previously published results, indicate that an object is encoded by a pattern of brain activation that is broadly distributed across the brain. The fact that it is possible to accurately identify the stimuli based on several different single regions alone raises a question of whether multiple brain regions redundantly encode the same information about the object, or whether each part of the brain encodes somewhat different information, reflecting its specialization. One way to compare the content of the neural representations in different regions is to compare the object confusion errors (incorrect first guesses) that the classifier makes when it uses input from various single regions, such as misidentifying a *hammer* as a *drill* based on only the left calcarine sulcus.

**Figure 7:**
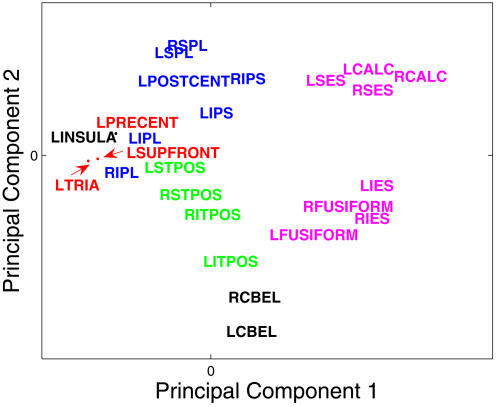
Brain regions in the space of the first two principal components of the compromise matrix based on the regions' confusion errors. The first principal component separates anterior and posterior regions, and accounts for 8% of the variance. The second principal component separates parietal and temporal regions, and accounts for 6% of the variance in the data. Region labels are color-coded by lobe, and are described in Table 1. The arrows are used to separate labels that are close to each other.

Suggestive evidence that the regions systematically differ from each other in terms of the confusion errors they generate was obtained from a principal components analysis (PCA) of the single regions' dissimilarity matrix. This matrix was constructed as a weighted average across participants (and captured 51% of the variability in the data, despite considerable variation among participants in their region-specific confusion matrices). When the confusion matrices generated by various single-region classifications were compared, a number of systematicities emerged, indicating that in fact the different regions were encoding different information. For example, a set of visual regions (CALC, FUSIFORM, IES, SES) were similar to each other with respect to the confusion errors that they generated, and they differed from a set of frontal regions (SUPFRONT, TRIA, PRECENT) in terms of their confusion errors. Figure 7 shows that a PCA of the dissimilarity of the regions' individual confusion errors produces separation of the regions, interpretable in terms of their anatomical locations, indicating that the brain activation that is used in identification differs qualitatively and systematically across regions, such as the posterior visual regions differing from frontal regions.

Another observation arising from this principal components analysis was that bilaterally homologous regions were similar to each other with respect to confusion errors, despite being physically distant from each other, suggesting that they represent and process rather similar information. This observation applies to most regions except for the frontal cortex, where the activation in the two hemispheres was more distinct and more left-lateralized. The PCA indicates that there are regularities to be explored, and other methods, such as repetition priming [23], [24], [25] may additionally be useful to further illuminate which object properties are represented in various regions.

### Commonality of neural representations across participants

Classifiers were trained on data from 11 of the 12 participants to determine if it was possible to identify object exemplars and categories in the held-out 12^th^ participant's data; this procedure was repeated for all participants. For object exemplars, reliable (p<0.001) identification accuracies were reached for eight out of twelve participants (unfilled bars in Figure 2). The highest exemplar identification rank accuracy obtained in this leave-one-participant-out method was 0.81 for one of the participants (compared to an accuracy of 0.53 from random predictions). The number of voxels for which the cross-participant object exemplar identification accuracy was greatest ranged from 50 to 2000 voxels, depending on the participant (Table S1).

For cross-participant identification of the object category, the highest rank accuracy obtained for one of the participants was 0.97 (the category was correctly identified on the first guess in 58 out of 60 object presentations) (unfilled bars in Figure 4). The classifier achieved reliable (p<0.001) accuracy in ten out of twelve participants. The mean accuracy across participants was 0.82 (SD = 0.09). The number of voxels for which the cross-participant category identification accuracy was greatest ranged from 10 to 2000 voxels, depending on the participant (Table S2). Voxel-by-voxel synchronization between individuals has been previously shown during movie watching [26]. The new result demonstrates the ability to identify the category of the object (and to some extent, the specific object) that a participant was viewing based on the neural signature derived from a set of other participants' activations. This finding indicates that much of the activation pattern that enables the identification of a cognitive state has a high degree of commonality across participants.

## Discussion

The two main conceptual advances offered by these findings are that there is an identifiable neural pattern associated with perception and contemplation of individual objects, and that part of the pattern is shared across participants. This neural pattern is characterized by a distribution of activation across many cortical regions, involving locations that encode diverse object properties. The results uncover the biological organization of information about visually depicted objects.

### Distributed representation

The fact that individual objects, and the categories they belong to, can be accurately decoded from fMRI activity in any of several regions indicates that there are multiple brain regions besides classical object-selective cortex that contain information about the objects and categories. These new findings raise the future research challenge of determining whether these multiple regions all contain similar information about the object (i.e., inter-region representational redundancy), or alternatively, whether each of the regions contains somewhat region-specific information about the object. The distributed patterns of activation evoked by objects which are being visualized include many of the parietal and prefrontal regions that contained diagnostic voxels in our study [7], [27], [28], [29]. The distributed activation pattern may reflect the distribution across cortical areas that are specialized for various types of object properties [7], [30], [31]. For example, the diagnostic voxels from the motor cortex that helped identify the hand *tools* may have represented the motor actions involved in the use of the tools. Similarly, parahippocampal voxels that were useful for identifying *dwellings* may have represented contextual information [32] about some aspect of dwellinghood that has earned this region the label of “parahippocampal place area” [22]. Similarly, other diagnostic regions presumably represented other types of visual and functional properties of the objects. An alternative characterization that is equally compatible with the empirical findings is that there are many ways in which we can think about, perceive, visualize, and interact with objects, for which different brain areas are differentially specialized. In this view, it is not just the isolated, intrinsic properties of the objects that are being represented, but also the different ways that we mentally and physically interact with the objects.

The information content within a number of individual anatomical regions is sufficient for exemplar and category identification, but the content of the representation appears to be somewhat different across regions. Comparison of the confusions in different regions suggests that despite the similarities in the identification accuracies provided by the various regions, anterior and posterior regions may represent different aspects of the objects, and that different brain regions provide the classifier with different kinds of information, likely corresponding to the different types of perceptual, motor, and conceptual processing that is performed in various brain regions.

### Commonality of the neural representation of object categories and exemplars across participants

The ability to identify object categories across participants reveals the striking commonality of the neural basis of this type of semantic knowledge. The neural invariances, in terms of the locations and activation amplitudes of common diagnostic voxels, emerged despite the methodological difficulty of normalizing the morphological differences among participants. The challenge of comparing the thoughts of different people has been met here in a very limited sense, although there always remains uncertainty about whether the information content corresponding to a diagnostic voxel's activity was the same across participants. Still, the new findings indicate that there is cross-participant commonality in the neural signature at the level of semantic property representations (and not just visual features).

The category and exemplar classification accuracies when training across participants were on average lower than when training within participants, indicating that a critical diagnostic portion of the neural representation of the categories and exemplars is still idiosyncratic to individual participants. There is apparently systematic activation within an individual (permitting better identification of that individual's cognitive state) that lends individuality to object representations.

Even though the classification accuracy was generally higher within as opposed to across participants, for a small number of participants (all of whom had low within-participant identification accuracies), identification based on training data from other participants actually resulted in higher accuracy than when training based on that participant's own data. In these few cases, the individual's idiosyncratic activation pattern may have been too variable over presentations to outperform the communal neural signature. These cases provide a demonstration of the remarkable power of the shared activation pattern to identify the thoughts of others.

## Supporting Information

Table S1 Identification accuracies of object exemplars based on the patterns of functional activity of that or other participants. Observed accuracies, number of voxels, and the p-value based on permutation distribution with 1,000 permutations are reported.
(0.04 MB DOC)

Table S2 Identification accuracies of object categories based on the patterns of functional activity of that or other participants. Observed accuracies, number of voxels, and the p-value based on permutation distribution with 1,000 permutations are reported.
(0.04 MB DOC)
