# Supplementary material for: Using fMRI Brain Activation to Identify Cognitive States Associated with Perception of Tools and Dwellings
Source: PLoS One. 2008 Jan 2;3(1):e1394. doi: 10.1371/journal.pone.0001394 (PMC2148074; doi:10.1371/journal.pone.0001394)
Supplement: Table S1 — Identification accuracies of object exemplars based on the patterns of functional activity of that or other participants. Observed accuracies, number of voxels, and the p-value based on permutation distribution with 1,000 permutations are reported. (0.04 MB DOC) [file pone.0001394.s001.doc]

|  | Within-participants | | | | | Across-participants | | | | | | |
| --- | --- | --- | --- | --- | --- | --- | --- | --- | --- | --- | --- | --- |
| Participants | Accuracy | Number of voxels | p-val | | Accuracy | | Number of voxels | | p-val | |  | |
| 1 | 0.72 | 200 | 0.00 | | 0.70 | | 200 | | 0.00 | |  | |
| 2 | 0.69 | 400 | 0.00 | | 0.75 | | 2000 | | 0.00 | |  | |
| 3 | 0.71 | 25 | 0.00 | | 0.71 | | 200 | | 0.00 | |  | |
| 4 | 0.88 | 100 | 0.00 | | 0.65 | | 2000 | | 0.04 | |  | |
| 5 | 0.82 | 25 | 0.00 | | 0.79 | | 100 | | 0.00 | |  | |
| 6 | 0.94 | 100 | 0.00 | | 0.79 | | 200 | | 0.00 | |  | |
| 7 | 0.72 | 200 | 0.00 | | 0.63 | | 1000 | | 0.01 | |  | |
| 8 | 0.91 | 75 | 0.00 | | 0.81 | | 200 | | 0.00 | |  | |
| 9 | 0.68 | 25 | 0.00 | | 0.62 | | 200 | | 0.00 | |  | |
| 10 | 0.81 | 400 | 0.00 | | 0.74 | | 200 | | 0.00 | |  | |
| 11 | 0.84 | 75 | 0.00 | | 0.68 | | 50 | | 0.00 | |  | |
| 12 | 0.59 | 75 | 0.05 | | 0.67 | | 2000 | | 0.01 | |  | |
| mean | 0.78 | 142 |  | | 0.71 | | 696 | |  | |  | |
| max | 0.94 | 400 |  | | 0.81 | | 2000 | |  | |  | |
| SD | 0.11 | 134 |  | 0.06 | | 823 | |  | |  | |  |
