# Supplementary material for: Using fMRI Brain Activation to Identify Cognitive States Associated with Perception of Tools and Dwellings
Source: PLoS One. 2008 Jan 2;3(1):e1394. doi: 10.1371/journal.pone.0001394 (PMC2148074; doi:10.1371/journal.pone.0001394)
Supplement: Table S2 — Identification accuracies of object categories based on the patterns of functional activity of that or other participants. Observed accuracies, number of voxels, and the p-value based on permutation distribution with 1,000 permutations are reported. (0.04 MB DOC) [file pone.0001394.s002.doc]

|  | Within-participants | | | | Across-participants | | | |
| --- | --- | --- | --- | --- | --- | --- | --- | --- |
| Participants | Accuracy | Number of voxels | p-val | Accuracy | | Number of voxels | p-val |  |
| 1 | 0.72 | 50 | 0.00 | 0.75 | | 2000 | 0.00 |  |
| 2 | 0.82 | 10 | 0.00 | 0.83 | | 1000 | 0.00 |  |
| 3 | 0.73 | 50 | 0.00 | 0.82 | | 400 | 0.00 |  |
| 4 | 1.00 | 50 | 0.00 | 0.90 | | 2000 | 0.00 |  |
| 5 | 0.88 | 25 | 0.00 | 0.73 | | 1000 | 0.01 |  |
| 6 | 0.97 | 10 | 0.00 | 0.87 | | 2000 | 0.00 |  |
| 7 | 0.88 | 100 | 0.00 | 0.87 | | 50 | 0.00 |  |
| 8 | 0.98 | 100 | 0.00 | 0.97 | | 2000 | 0.00 |  |
| 9 | 0.85 | 10 | 0.00 | 0.63 | | 10 | 0.06 |  |
| 10 | 0.87 | 25 | 0.00 | 0.83 | | 75 | 0.00 |  |
| 11 | 0.97 | 50 | 0.00 | 0.87 | | 1000 | 0.00 |  |
| 12 | 0.78 | 10 | 0.00 | 0.78 | | 75 | 0.00 |  |
| mean | 0.87 | 41 |  | 0.82 | | 968 |  |  |
| max | 1.00 | 100 |  | 0.97 | | 2000 |  |  |
| SD | 0.10 | 33 |  | 0.09 | | 850 |  |  |
